# Axillary accessory breast cancer with persistent leftsuperior vena cava: A case report and treatment controversy

**DOI:** 10.1016/j.ijscr.2020.05.038

**Published:** 2020-06-20

**Authors:** Jun Zhang, Weidong Zhang, Meilin Min, Yunbo Pan

**Affiliations:** a Wuxi Hospital Affiliated to Nanjing University of Chinese Medicine, Wuxi, China; b Wuxi Higher Health Vocational Technology School, Wuxi, China

**Keywords:** Accessory breast cancer, Treatment controversy, Case report, Persistent left superior vena cava

## Abstract

**Introduction:**

Axillary accessory breast cancer and persistent left superior vena cava (PLSVC) are rare clinically. Many controversial treatments for accessory breast cancer are worth discussing and learning.

**Presentation of the case:**

A 48-year-old woman presented with biopsy histopathology confirmed. Right axillary mass biopsy pathology showed mucinous adenocarcinoma of accessory breast. She concerned that the axillary accessory breast cancer was more likely to metastasize and unsure about whether to remove the breast. She accepted extended right axillary accessory breast resection plus ipsilateral axillary lymph node dissection (ALND) and received chemotherapy. She was found to have a PLSVC before chemotherapy.

**Discussion:**

Is there a need to remove the breast and perform ALND during axillary accessory breast cancer surgery? Is sentinel lymph node biopsy (SLNB) appropriate for axillary accessory breast cancer surgery? Can negative SLNB for axillary accessory breast cancer avoid ALND? Does accessory breast cancer without axillary lymph node metastasis require local radiotherapy? Does PLSVC impact the use of peripherally inserted central catheters (PICC) tubes during chemotherapy? Patients with accessory breast cancer without breast invasion should undergo local extended resection and ALND. SLNB for accessory breast cancer cannot instead of ALND. We recommend routine axillary radiotherapy after accessory breast cancer surgery. If it is determined that the tip of PICC is not in the coronary sinus of PLSVC, PLSVC does not affect chemotherapy.

**Conclusion:**

Many treatment strategies for accessory breast cancer require more evidence from evidence-based medicine. It is imperative to conduct multi-center accessory breast cancer research.

## Highlights

• Accessory breast cancer with persistent left superior vena cava(PLSVC) is rare.
• The treatments of axillary accessory breast cancer should attract our attention.
• Patients with accessory breast cancer without breast invasion should undergo local enlarged resection and axillary lymph node dissection (ALND).
• We recommend routine axillary radiotherapy after accessory breast cancer surgery.
• It is imperative to organize multi-center accessory breast cancer research.

## 1 Introduction

Accessory breast can occur at any part of the breast baseline from the axilla, chest, abdomen to groin [1]. Benign and malignant diseases can occur in accessory mammary glands as well as the breast [2]. Accessory breast cancer is rare clinically, and the prognosis of accessory breast cancer is reported to be worse than that of breast cancer [3]. Many controversial treatments for accessory breast cancer are worth discussing and learning. It is reported that the incidence of persistent left superior vena cava(PLSVC) in congenital heart disease is 1.7%–4.3% [4]. The work has been reported in line with the SCARE criteria [5]. The organization that manages the patient is a public hospital.

## 2 Report of the case

**Fig. 1:**
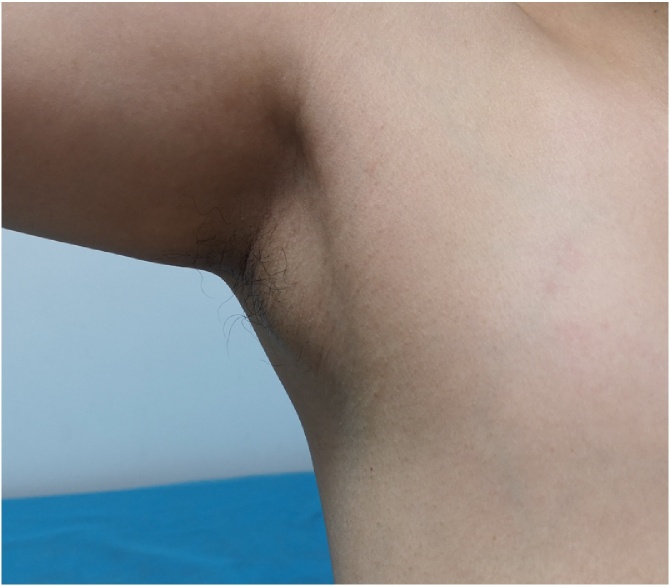
Accessory breast.

A 48-year-old woman was found with a right axillary mass of about 1.5 cm × 1.0 cm in size one year ago. At that time, it was misdiagnosed as an enlarged lymph node without any treatment, and then the right axillary mass gradually increased. There was no family history of breast carcinoma. The patient had four abortions. She had a breastfeeding history of 4 months. Physical examination showed that the right axillary para-breast (Fig. 1) had a soft texture, clear margin and no vice nipple. A mass of about 2.5 cm × 1.5 cm could be touched, with a hard texture, unclear margin, poor mobility, no adhesion to the skin. No mass was touched by both breast.

**Fig. 2:**
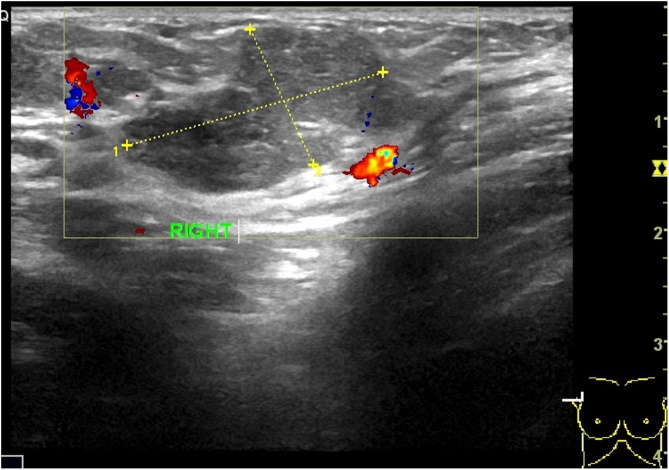
Doppler ultrasonography.

**Fig. 3:**
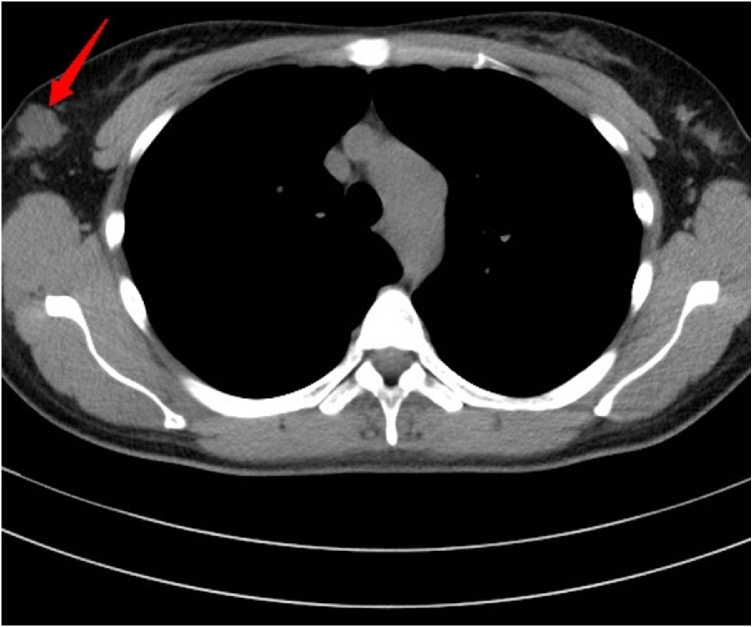
Chest CT.

The right axillary hypoechoic mass (Fig. 2) was revealed by subaxillary color Doppler ultrasound. Chest Computed Tomography plain scan showed the right axillary space was occupied (Fig. 3), and there was no obvious abnormality in both lungs. Double breast MRI(Magnetic Resonance Imaging) showed that no mass was found in the right breast, and multiple cystic signal cysts were found in the left breast. The results of tumor markers were normal. Right axillary mass biopsy pathology showed mucinous cancer of accessory breast.

**Fig. 4:**
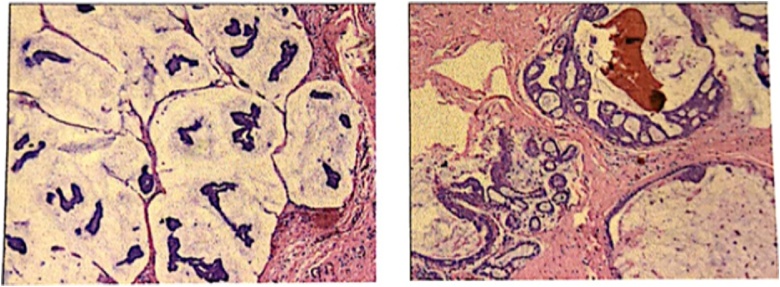
Pathological picture.

**Fig. 5:**
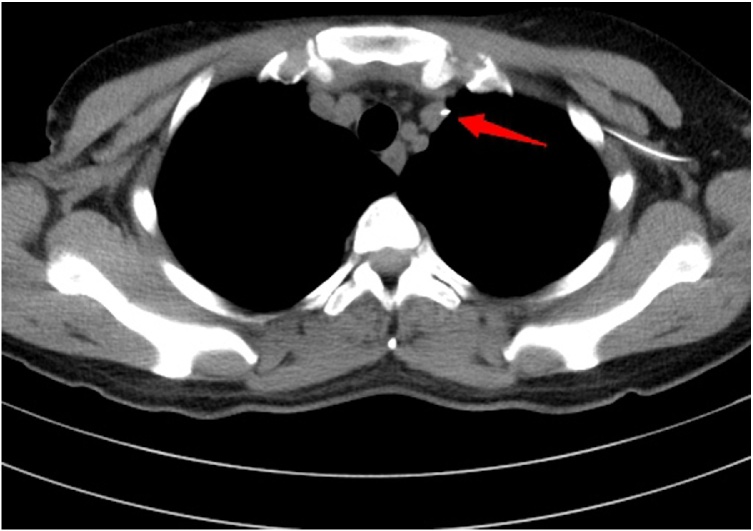
Permanent left superior vena cava.

**Fig. 6:**
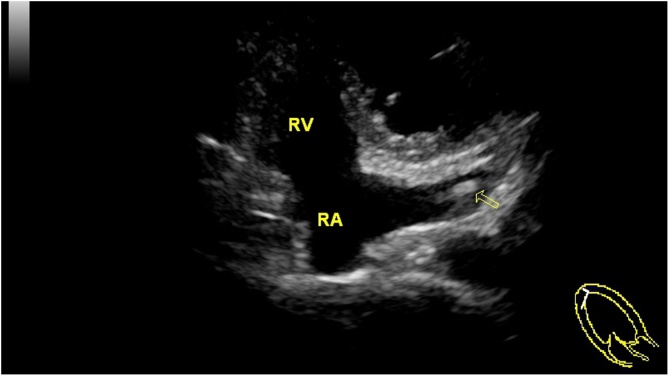
Cardiac color Doppler.

Then the patient underwent extended right axillary accessory breast resection plus ipsilateral axillary lymph node dissection (ALND). There were two adjacent masses in the accessory breast tissue with sizes of 2.0 cm × 1.0 cm and 1.0 cm × 0.8 cm respectively. There was white sticky substance in the mass. There was a clear boundary between the accessory breast tissue and the breast tissue. Postoperative pathology (Fig. 4) showed right axillary mucinous carcinoma, which was locally micropapillary and surrounding (surround by) medium-grade intraductal cancer. The vascular and nerve had no cancer invasion, the axillary lymph node had no cancer metastasis and the skin incision margin had no cancer involvement. The immunohistochemistry of the mucinous cancer showed 70% estrogen receptor-positive, 80% progesterone receptor-positive, Her2/neureceptor-negative and 30% antigen KI67-positive.The pathological stage was T2N0M0(IIA), the molecular typing was Luminal B. After surgery, the patient received four rounds of chemotherapy. Before chemotherapy, peripherally inserted central catheters (PICC) catheterization was performed on the left upper limb. Chest X-ray showed that PICC was located on the left side of the heart. Chest CT (Fig. 5) and color Doppler echocardiography (Fig. 6) found the existence of PLSVC. After chemotherapy, the patient received endocrine therapy and refused radiotherapy.

## 3 Discussion

Accessory breast cancer is very rare, accounting for 0.3%–0.6% of all breast cancers, often manifesting as axillary mass [6]. In China, one group report was from the Tumor Hospital Affiliated to Tianjin Medical University. The incidence was about 0.15% [7] of breast cancer in the same period. Therefore, there is currently a lack of clinical studies involving large sample sizes of accessory breast cancer in clinical practice.

The prognosis of accessory breast cancer is reported to be worse than that of breast cancer. The 5-year survival rate of accessory breast cancer reported by Cancer Hospital of Chinese Academy of Medical Sciences was 41.7% (compared with 71.0% of breast cancer in the same period) [3]. The total 5-year survival rate of accessory breast cancer reported by Tianjin Cancer Hospital was 35.3% (lower than 66.8% of breast cancer in the same period, P < 0.05) [8]. Therefore, the treatments of axillary accessory breast cancer should attract our attention.

Is there a need to remove the breast and perform ALND during axillary accessory breast cancer surgery? Made et al. [9] reported that the surgical scope of accessory breast cancer could be reduced by drawing on the experience of breast-conserving surgery for breast cancer. Unless there is clear evidence that accessory breast tumors are closely linked to the breast, the ipsilateral breast should be preserved. Evans et al. [10] reported that radical or modified radical mastectomy was used in the operation of accessory breast cancer. But compared with the combination of local resection and axillary lymphadenectomy, there was no significant difference in survival period between the two groups. Most Chinese scholars advocate that the surgical method of accessory breast cancer is enlarged excision of tumors and ipsilateral ALND, and ipsilateral mastectomy can be performed simultaneously in those with cancer infiltration in the ipsilateral breast. In the case of no difference in survival, mastectomy has a great impact on the patient's appearance and also affects the patient's self-confidence. Therefore, patients with accessory breast cancer without breast invasion should preserve breast and undergo local extended resection and ALND.

Is sentinel lymph node biopsy (SLNB) appropriate for axillary accessory breast cancer surgery? Can the negative SLNB for axillary accessory breast cancer avoid ALND? There is a consensus that negative SLNB in early stage breast cancer can replace ALND. However, the location of the axillary accessory breast cancer is relatively special. At present, no report has been made on the lymphatic return of the axillary accessory breast cancer and SLNB for axillary accessory breast cancer. If SLNB is performed for axillary accessory breast cancer, the question is whether the tracer is injected on the vice nipple or the breast areola? Lymph nodes marked by injection of tracer in the areola and in the para-milk may be inconsistent. Therefore, SLNB for axillary accessory breast cancer needs further research to confirm. We do not recommend SLNB for accessory breast cancer instead of ALND.

Does accessory breast cancer without axillary lymph node metastasis require local radiotherapy? In clinical practice, there are no specific guidelines of radiotherapy for accessory breast cancer. Some people believe that local radiotherapy is not required after surgery for accessory breast cancer without axillary lymph node metastasis.

However, some scholars [11] believe that accessory breast cancer mostly occurs near the axilla, lymph node metastasis is more likely to occur early, and the indications for radiotherapy should be relaxed appropriately. It has been reported that patients with accessory breast cancer need conventional radiotherapy after surgery to reduce the risk of local recurrence [12]. Therefore we recommend routine radiotherapy after accessory breast cancer surgery.

The persistent left superior vena cava(PLSVC) is due to the unclosed left anterior vein and left venous catheter during embryonic development [13], and it is the commonest thoracic venous anomaly. Does PLSVC impact the use of PICC tubes during chemotherapy? The left superior vena cava often connects to the coronary sinus and opens to the right atrium, often accompanied by dilatation of the coronary sinus. If the PICC tube is heterotopic in the coronary sinus, the pressure changes in the coronary sinus during infusion and the chemical damage of the vascular wall caused by highly stimulating chemotherapeutics can lead to serious complications such as arrhythmia, coronary sinus thrombosis, and even angina pectoris and myocardial necrosis [14]. Therefore, if it is determined that the tip of PICC is not in the coronary sinus of PLSVC, PLSVC does not affect chemotherapy.

## 4 Conclusion

Axillary accessory breast cancer is relatively rare. Many treatment strategies for axillary accessory breast cancer require more evidence from evidence-based medicine. It is imperative to conduct multi-center accessory breast cancer research.

## Declaration of Competing Interest

The authors declare that they have no conflict of interest.

## Sources of funding

The study has funding from Scientific Research Project of Wuxi Health and Family Planning Commission (MS201841).

Jun Zhang is the main initiator of the research. He participated in the conception of the paper, data collection, writing and deciding which journal to submit.

## Ethical approval

The study passed the ethical review of IRB of Wuxi Hospital of Traditional Chinese Medicine, and the ethical review approval number is LW2020052001.

## Consent

Written informed consent was obtained from the patient for publication of this case report and accompanying images.

## Author contribution

1. Jun Zhang: Conceptualization; Data curation; Funding acquisition; Investigation; Methodology; Resources; Writing - original draft.
2. Weidong Zhang: Investigation; Resources; Data curation; Writing - review & editing, Validation
3. Meilin Min: Data curation; Investigation; Methodology.
4. Yunbo Pan: Investigation; Writing-review & editing.

## Registration of research studies

1. Name of the registry: NA.
2. Unique identifying number or registration ID: NA.
3. Hyperlink to your specific registration (must be publicly accessible and will be checked): NA.

## Guarantor

The Guarantor is Jun Zhang.

## Provenance and peer review

Not commissioned, externally peer-reviewed.
